# Amino Acid Availability Controls TRB3 Transcription in Liver through the GCN2/eIF2α/ATF4 Pathway

**DOI:** 10.1371/journal.pone.0015716

**Published:** 2010-12-21

**Authors:** Valérie Carraro, Anne-Catherine Maurin, Sarah Lambert-Langlais, Julien Averous, Cédric Chaveroux, Laurent Parry, Céline Jousse, Daima Örd, Tõnis Örd, Pierre Fafournoux, Alain Bruhat

**Affiliations:** 1 INRA, UMR 1019 Nutrition Humaine, Saint Genès Champanelle, France; 2 Université Clermont 1, UFR Médecine, UMR 1019 Nutrition Humaine, Clermont-Ferrand, France; 3 Goodman Cancer Centre, McGill University, Montréal, Canada; 4 Estonian Biocentre, Tartu, Estonia; Chinese University of Hong Kong, Hong Kong

## Abstract

In mammals, plasma amino acid concentrations are markedly affected by dietary or pathological conditions. It has been well established that amino acids are involved in the control of gene expression. Up to now, all the information concerning the molecular mechanisms involved in the regulation of gene transcription by amino acid availability has been obtained in cultured cell lines. The present study aims to investigate the mechanisms involved in transcriptional activation of the *TRB3* gene following amino acid limitation in mice liver. The results show that *TRB3* is up-regulated in the liver of mice fed a leucine-deficient diet and that this induction is quickly reversible. Using transient transfection and chromatin immunoprecipitation approaches in hepatoma cells, we report the characterization of a functional Amino Acid Response Element (AARE) in the *TRB3* promoter and the binding of ATF4, ATF2 and C/EBPβ to this AARE sequence. We also provide evidence that only the binding of ATF4 to the AARE plays a crucial role in the amino acid-regulated transcription of *TRB3*. In mouse liver, we demonstrate that the GCN2/eIF2α/ATF4 pathway is essential for the induction of the *TRB3* gene transcription in response to a leucine-deficient diet. Therefore, this work establishes for the first time that the molecular mechanisms involved in the regulation of gene transcription by amino acid availability are functional in mouse liver.

## Introduction

Mammals have evolved a wide range of adaptative mechanisms to detect and respond to fluctuations in dietary nutrients. In particular they have to precisely regulate amino acid homeostasis taking into account two important characteristics of amino acid metabolism: (i) multicellular organisms are unable to synthesize all amino acids and (ii) there is no important dispensable amino acid store. Amino acidemia can be markedly affected by physiological or pathological conditions such as protein under-nutrition, imbalanced diet and various forms of stress (trauma, sepsis, etc.). Consequently, in order to adapt to amino acid availability, mammals have to adjust several physiological functions. One of the signal transduction pathways that is triggered in response to protein or amino acid starvation is referred to as the GCN2/eIF2α/ATF4 pathway [1]. The initial step in this pathway is the activation by uncharged tRNAs of the GCN2 kinase which phosphorylates the α subunit of translation initiation factor eIF2 (eIF2 α on serine 51 [2], [3]. This phosphorylation decreases the translation of most mRNAs by inhibiting the delivery of the initiator Met-tRNA_i_ to the initiation complex. However, eIF2α phosphorylation also favors increased translation of a selected number of mRNAs, including that coding for the activating transcription factor 4 (ATF4). Once induced, ATF4 directly or indirectly induces transcription of a subset of specific target genes [4], [5].

In cultured cell lines, several amino acid-responsive genes such as *ASNS* (Asparagine synthetase) [6], [7], [8] and *CHOP* (C/EBP homologous protein) [9], [10], [11] have been reported to contain AAREs (Amino Acid Response Elements) that mediate the enhanced transcription and function as enhancer elements [10]. The AARE sites have a 9 bp core element (5′-^A^/_G_TT^G^/_T_CATCA-3′) but the sequences can differ by one or two nucleotides between genes. It is now established that in amino acid-starved cells, a multiproteic complex is bound to the AARE sequences including a number of regulatory proteins such as ATF4 [7], [12], [13], CCAAT/enhancer binding protein β (C/EBPβ) [14], activating transcription factor 2 (ATF2) [15] or activating transcription factor 3 (ATF3) [13]. These factors are involved in either inducing or repressing transcription of target genes in response to amino acid starvation. Importantly, all of the known AARE sites bind ATF4, a master regulator of a number of amino acid-regulated genes. The binding activity and the role of other AARE binding factors appear to vary according to the AARE sequence and chromatin structure. For example, *CHOP* and *ATF3* sequences also bind ATF2 whereas *ASNS* and *SNAT2* sites do not [10], [11], [12], [13]. Chromatin immunoprecipitation (ChIP) experiments have revealed that there is a highly coordinated time-dependent program of interaction between a precise set of ATF subfamily members and coactivators leading to transcriptional activation of amino acid-regulated genes [8], [15].

Tribbles homolog 3 (TRB3) (also known as TRIB3, NIPK or SKIP3) is a pseudokinase which interacts with several transcription factors [16], [17], [18], protein kinases [19], [20] and other proteins [21], [22] and has been implicated in the control of stress response, cell viability and metabolic processes such as glucose or lipid metabolism. In particular, TRB3 has been identified as a feedback inhibitor of ATF4 involved in the transcriptional control of stress-regulated genes [16], [23], [24]. TRB3 has also been linked to pathophysiological conditions, including insulin resistance [19], [25], [26], cardiovascular disease [27] and diabetes [28]. *TRB3* is an inducible gene whose expression is modulated by metabolic stresses including endoplasmic reticulum stress [17], [29] and nutrient stress [24], [30] and by insulin [31], [32], [33]. In the context of gene regulation by amino acid starvation, the role of TRB3 as a feedback inhibitor of ATF4 in amino acid-regulated transcription has been previously studied [24]. It was shown that (i) TRB3 overexpression inhibits *CHOP* and *ASNS* induction by leucine starvation, (ii) TRB3 is associated with ATF4 in the protein complex bound to the AARE sequence. It has been also documented that TRB3 is involved in the control of the basal level of gene expression under control/unstarved conditions [24]. At the level of gene expression, TRB3 appeared to be one of the most amino acid-induced transcripts in cultured cell lines. However, the mechanisms by which amino acids control *TRB3* expression remained to be investigated.

Up to now, all the published data concerning the molecular mechanisms involved in the regulation of gene transcription by amino acid availability have been obtained in cultured cell lines. No result concerning these mechanisms was obtained *in vivo* in mammalian tissues. The present study aims to investigate the molecular mechanisms involved in the transcriptional activation of the *TRB3* gene following amino acid limitation in mouse liver. Our results show that the *TRB3* gene is up-regulated in the liver of mice fed a leucine-deficient diet. Using transient transfection and chromatin immunoprecipitation approaches in hepatoma cells, we report the characterization of an Amino Acid Response Element (AARE) in the *TRB3* promoter and the binding of ATF4, ATF2 and C/EBPβ to this AARE sequence. We provide also evidence that the binding of ATF4 to the AARE plays a crucial role in the amino acid-regulated transcription of *TRB3*. In mouse liver, we demonstrate that the GCN2/eIF2α/ATF4 pathway is essential for induction of the *TRB3* gene transcription in response to a leucine-deficient diet. Therefore, this work establishes that the molecular mechanisms involved in the regulation of gene transcription by amino acid availability are functional in mouse liver.

## Results

### Induction of hepatic *TRB3* expression in response to one essential amino acid starvation

**Figure 1:**
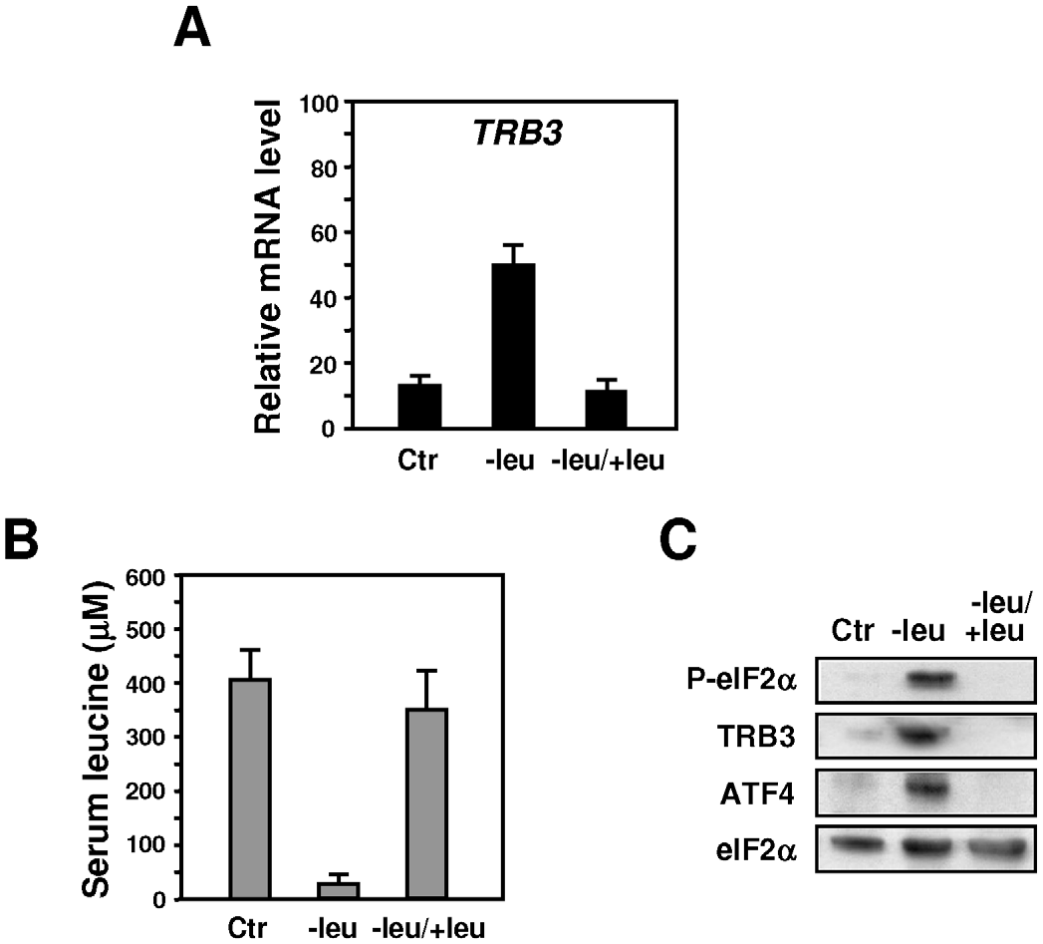
Induction of TRB3 mRNA in the liver of mice fed a diet lacking leucine vs control diet. Mice were fed for 2 h either control (Ctr) or leucine-deficient (−leu) diets or refed for 2 h on the control diet after a 2 h-consumption of the leucine-deficient diet (−leu/+leu) prior to liver mRNA and protein or plasma leucine levels measurement. (A) Total hepatic RNA from mice fed the indicated chow was subjected to qRT-PCR analysis for mRNA content as indicated. (B) Plasma leucine levels (µM) from mice fed as indicated above. (C) Liver protein extracts from mice fed the indicated chow were prepared as described under “Materials and Methods” and immunoblots against eIF2α phosphorylated on serine 51, ATF4 and TRB3 were performed.

We first investigated the induction of *TRB3* in the liver of mice fed a leucine-deficient diet for 2 h. When compared with the control diet, the consumption of chow lacking leucine leads to a large induction of *TRB3* expression in the liver (Figure 1A) while a sharp decrease of leucine level (from 370 to 33 µM) was observed in the plasma (Figure 1B). Protein analysis shows that leucine deprivation induced phosphorylation of eIF2α, and expression of ATF4 and TRB3 (Figure 1C). Refeeding 2 h a control diet after a 2 h-consumption of a leucine-deficient diet was associated with a rapid increase of leucine plasma level and with marked decreases in eIF2α phosphorylation and ATF4, TRB3 expression.

**Figure 2:**
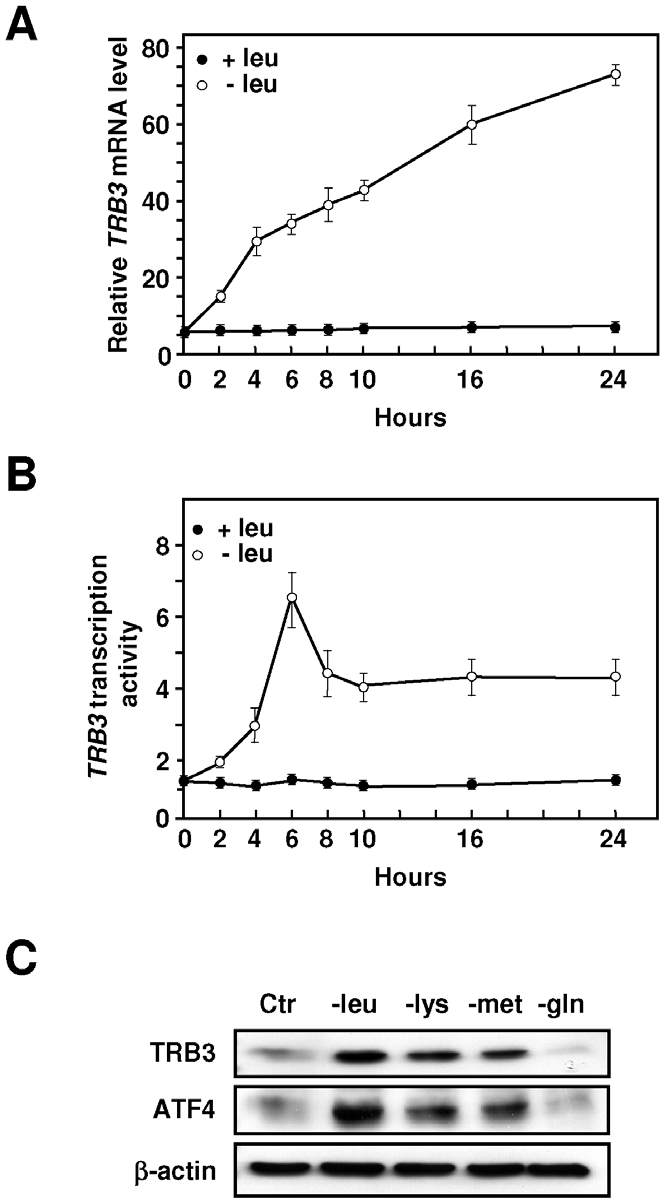
Measurement of *TRB3* expression in HepG2 cells following leucine starvation. (A) and (B) HepG2 cells were incubated for 0–24 h either in control (+leu) or leucine-free medium (−leu) and harvested after the indicated incubation times. The steady-state mRNA levels for TRB3 were assayed using primers within the protein coding sequence whereas the measurement of TRB3 pre-mRNA was determined using primers spanning the intron 2-exon 3 junction as described under “Materials and Methods”. (**C**) HepG2 cells were incubated either in control (Ctr) or in a medium devoid of one individual amino acid (leucine, lysine, methionine or glutamine) for 16 h and western blot analysis of TRB3, ATF4 and β-actin was performed as described in the “Materials and Methods” section.

To further characterize the mechanism of transcriptional activation of *TRB3* following amino acid starvation, cultured hepatocyte-derived HepG2 cells were used. HepG2 cells were first incubated in leucine-free medium for 0–24 h and the mRNA levels for TRB3 were measured (Figure 2A). An initial increase in TRB3 mRNA content was observed after 2 h of leucine deprivation and reached a value of about 14-times higher than the control after 24 h. To investigate whether transcription contributed to the increase of TRB3 mRNA in HepG2 cells, the transcription activity of *TRB3* was measured by analysing the synthesis of hnRNA [34]. Within 2 h after leucine removal, transcription was increased and continued to rise until reaching a peak of six times the control value at 6 h, and then rapidly declined (Figure 2B). These results show that increased transcription contributes to the elevation in TRB3 mRNA following amino acid limitation. The difference in absolute magnitude between the increase in transcription rate and the steady-state mRNA could be explained by the amino acid-dependent stabilization of the TRB3 mRNA as described for some other amino acid-regulated genes [1], [35]. Furthermore, the effects on TRB3 protein expression were checked for some other individual amino acids. Figure 2C shows that starvation for essential amino acids such as lysine and methionine enhanced TRB3 expression whereas glutamine starvation (a non-essential amino acid) had no significant effect. This experiment also showed that ATF4 expression is induced by leucine, lysine or methionine starvation in HepG2 cells.

### Identification of a functional Amino Acid Response Element in the *TRB3* promoter

**Figure 3:**
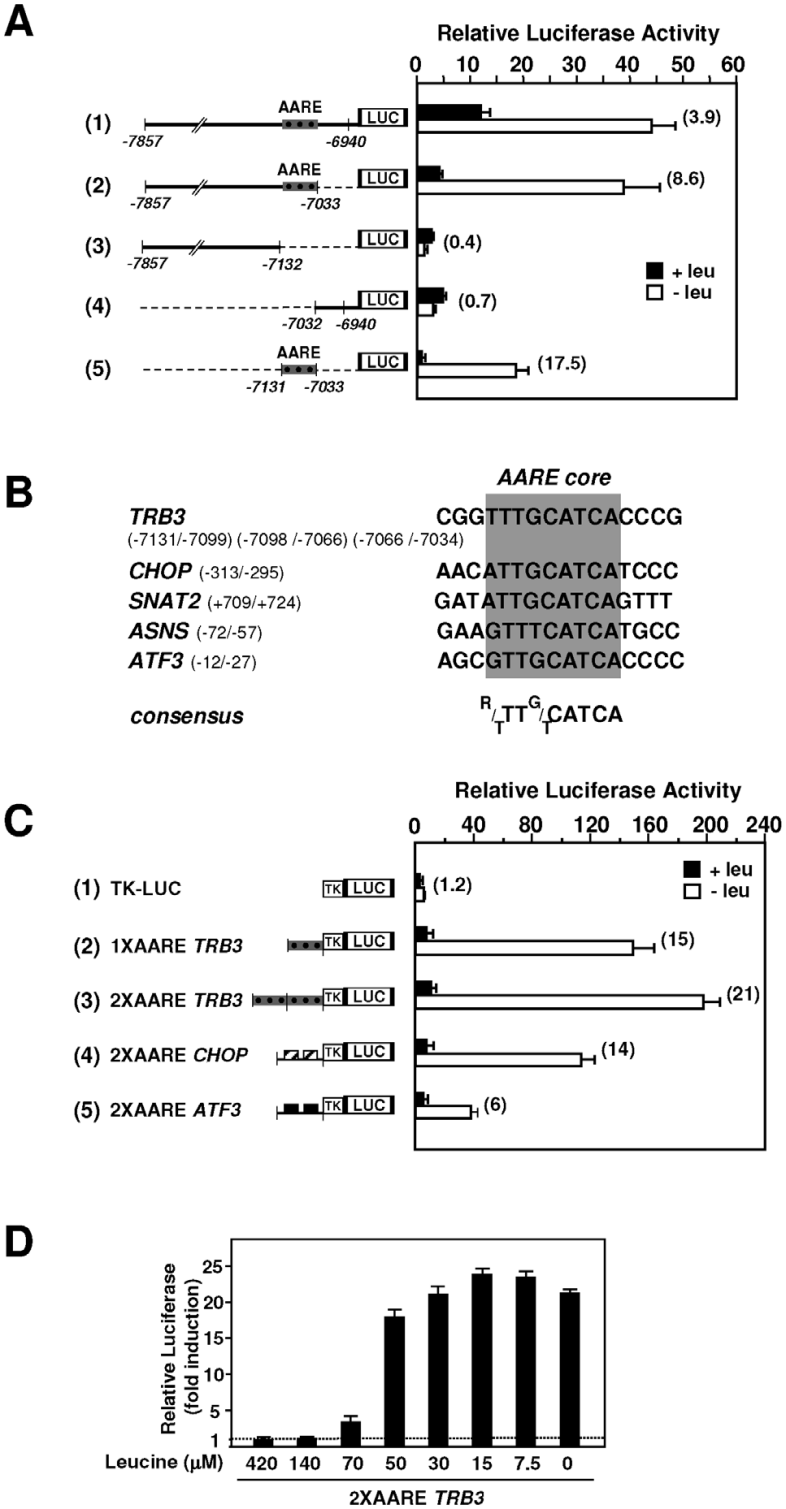
Identification of the AARE regulating activation of transcription of *TRB3* in response to amino acid starvation in HepG2 cells. (A) HepG2 cells were transiently transfected with LUC constructs containing internal deletions of the *TRB3* promoter as described under “Materials and Methods”. Twenty-four hours after transfection, cells were incubated for 24 h in control DMEM F12 (+leu) or in DMEM F12 lacking leucine (−leu) and assayed for LUC activity. The relative fold induction, defined as the ratio of the relative LUC activity of leucine-starved cells to unstarved cells, is indicated in parentheses to the right of the bars. (B) Sequence comparison of the *TRB3* 33-bp repeat (−7131/−7099, −7098/−7066, −7066/−7034) with the *CHOP* AARE (−313/−295), the *SNAT2* AARE (+709/+724), the *ASNS* NSRE-1 (−57/−72) and the *ATF3* AARE (−12/−27). The position of the minimum AARE core sequence is boxed in grey. The resulting minimum consensus sequence is shown at the bottom. (C) HepG2 cells were transiently transfected with LUC constructs containing one or two copies of the *TRB3* AARE (−7131/−7033) or two copies of the *CHOP* AARE or *ATF3* AARE inserted 5′ to the *TK* promoter. Twenty-four hours after transfection, cells were incubated for 24 h in DMEM F12 (+leu) or in DMEM F12 lacking leucine (−leu) and assayed for LUC activity. (D) HepG2 cells were transiently transfected with a 2X*TRB3*AARE-LUC construct. Twenty-four hours after transfection, cells were incubated for 24 h in DMEM F12 (420 µM) or in DMEM F12 containing the indicated leucine concentration and assayed for LUC activity.

In the 5′-upstream region of the human *TRB3* gene, from −7131 to −7033, three identical tandemly arranged repeats each consisting of 33-bp were previously described [17], [29]. This promoter region was reported to contain an endoplasmic reticulum (ER) stress response element. To investigate further the contribution of these repeats to the amino acid-dependent increase in TRB3 transcription, a series of deletions in the promoter was created and fused to the Firefly luciferase reporter gene, in an assay for the response to amino acid deprivation. Figure 3A shows that a −7857 to −6940 promoter fragment (row 1) was able to mediate a level of induced response to amino acid deprivation which is consistent with the 4-fold increase in the transcription rate (see Figure 2B). Further *TRB3* promoter deletions can be divided into two groups according to their level of amino acid inducibility. The first group includes two deletions that produced high levels of amino acid inducibility (rows 2 and 5) while the second group of deletions led to complete loss of amino acid inducibility (rows 3 and 4). These finding demonstrate that the 99-bp *TRB3* promoter region from −7131 to −7033 including the three tamdemly arranged 33-bp-long repeats is essential for amino acid regulation.

Close inspection of the sequence of the middle part of each 33-bp repeat reveals similarity with the Amino Acid Response Elements (AARE) core sequence identified in other amino acid regulated genes such as *CHOP*, *ASNS*, system A amino acid transporter (*SNAT2*) and *ATF3* (Figure 3B). To examine whether this 99-bp *TRB3* promoter sequence could, by itself, render a heterologous promoter amino acid responsive, one or two copies of this segment were cloned 5′ of the minimal herpes simplex virus promoter for thymidine kinase (TK). As shown in Figure 3C, a single copy of the *TRB3* promoter sequence was able to regulate the basal promoter in response to leucine starvation (row 2). Furthermore, the leucine starvation-induced activity was more strongly enhanced by the presence of two copies of this *TRB3* sequence (row 3) and the induced response to amino acid deprivation was greater than those observed with two copies of *CHOP* or *ATF3* AARE sequences (rows 4 and 5). Figure 3D shows that the transcriptional activity from the *TRB3* AARE was enhanced by a decrease in leucine concentration in a dose-dependent manner in the range of those observed in the blood of leucine-starved animals (see Figure 1B). Altogether, these results demonstrate that the 99-bp sequence in the *TRB3* promoter can be considered as an AARE that can be efficient at a physiological leucine concentration.

### The induction of *TRB3* transcription in response to amino acid limitation is dependent of GCN2 and independent of PERK

**Figure 4:**
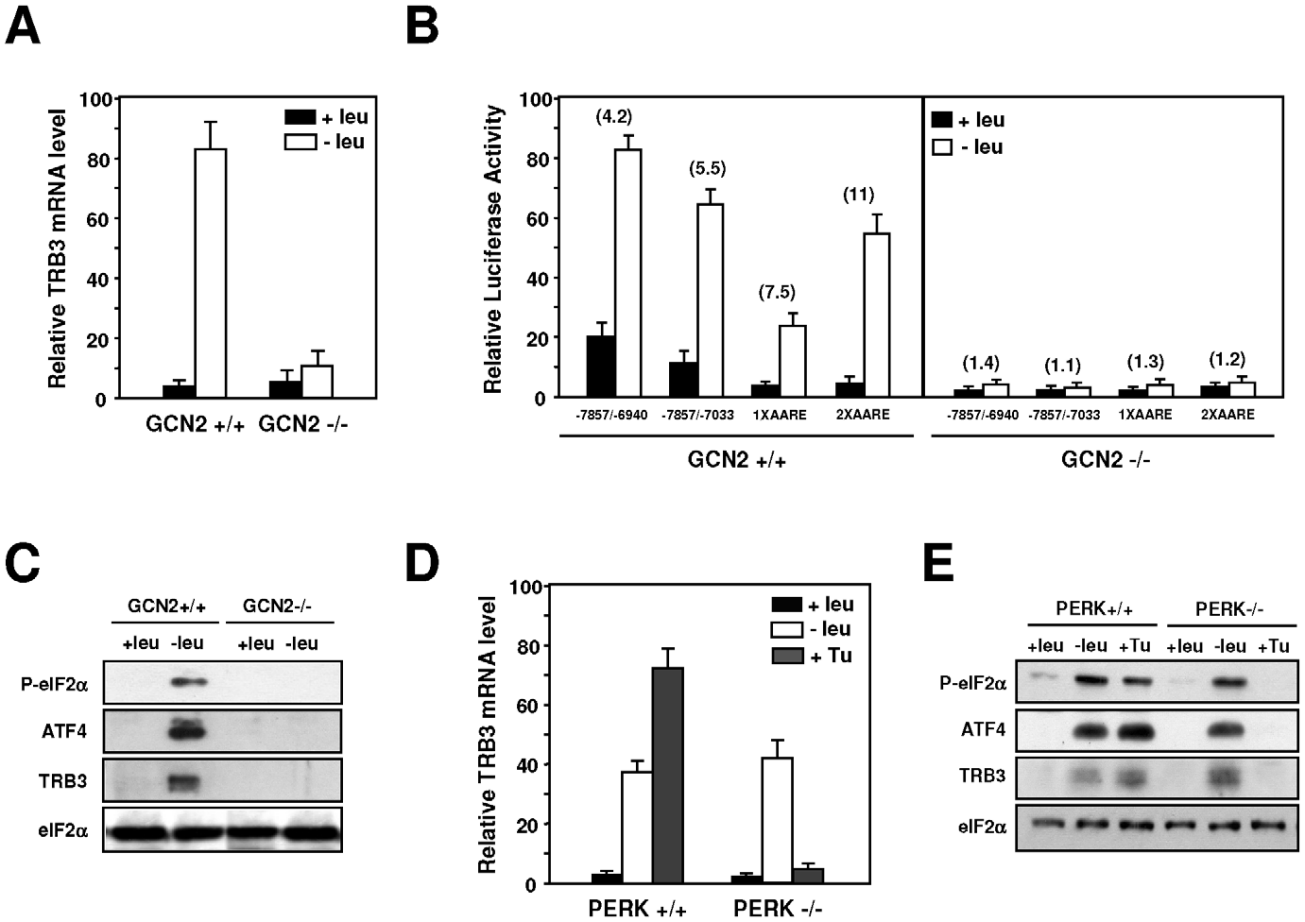
Role of GCN2 and PERK in the transcriptional regulation of *TRB3* following amino acid starvation. (A) GCN2 +/+ or GCN2 −/− MEFs were incubated either in control (+leu) or leucine-free medium (−leu) and harvested after 4 hours and total RNA was analyzed for *TRB3* mRNA content as described under “Materials and Methods”. The graphs show means ± S.E.M. of three independent experiments. (B) GCN2 +/+ or GCN2 −/− MEFs were transiently transfected with four amino acid responsive luciferase constructs as described under “Materials and Methods”. Twenty-four hours after transfection, cells were incubated for 24 h in control DMEM F12 (+leu) or in DMEM F12 lacking leucine (−leu) and then were assayed for LUC activity. The relative fold induction, defined as the ratio of the relative LUC activity of leucine-starved cells to unstarved cells, is indicated in parentheses. (C) Phosphorylation of eIF2α on serine 51, ATF4, TRB3 and eIF2α protein content were analyzed by western blots as described in the “Materials and Methods” section. (D) PERK +/+ or PERK −/− MEFs were incubated for 4 hours either in control (+leu) or leucine-free medium (−leu) or for 4 hours in medium containing 0.5 µg/ml of tunicamycin (+Tu). Total RNA was analyzed for *TRB3* mRNA content as described under “Materials and Methods”. The graphs show means ± S.E.M. of three independent experiments. (E) Phosphorylation of eIF2α on serine 51, ATF4, TRB3 and eIF2α protein contents were analyzed by western blots as described in “Materials and Methods” section.

The GCN2 kinase has been shown to be involved in the up-regulation of a set of specific genes in response to amino acid starvation [36]. We first examined the role of GCN2 in the amino acid regulation of *TRB3* transcription in MEF cells. The effect of leucine starvation was measured on both TRB3 mRNA content and *TRB3* promoter-dependent transcription in GCN2-deficient MEFs and in the corresponding wild-type cells. MEF cells were transfected with four amino acid responsive luciferase constructs (see rows 1 and 2 of Figure 3A and rows 2 and 3 of Figure 3C) and then incubated either in control or leucine-starved medium. Lack of GCN2 abolished the response of *TRB3* transcription to leucine depletion: the amino acid inducibility of both TRB3 mRNA (Figure 4A) and *TRB3* AARE (Figure 4B) were completely lost. Protein analysis confirms that the induction of eIF2α phosphorylation was completely lost in MEFs deficient in GCN2 (Figure 4C). These results demonstrate that GCN2 plays a critical role in the activation of *TRB3* promoter in response to amino acid starvation in MEF cells.

*TRB3* was previously described as an ER stress-inducible gene [17], [29]. To address directly the role of PERK, the ER stress-activated eIF2α kinase, in the transcriptional response of *TRB3* to leucine starvation, PERK-deficient MEFs were used. In PERK+/+ MEFs (Figure 4D), *TRB3* exhibited a normal response to leucine starvation and to an agent (tunicamycin) that induces ER stress. Lack of PERK resulted in a complete loss in the TRB3 mRNA inducibility by tunicamycin but did not affect the mRNA induction level by leucine starvation. Protein analysis confirms that inductions of eIF2α phosphorylation and TRB3, ATF4 expression were still observed in leucine-starved PERK−/− cells (Figure 4E). On the other hand, eIF2α remained unphosphorylated and TRB3 and ATF4 levels did not increase when these cells were treated with tunicamycin. Thus, PERK is not required for the *TRB3* induction following leucine starvation.

**Figure 5:**
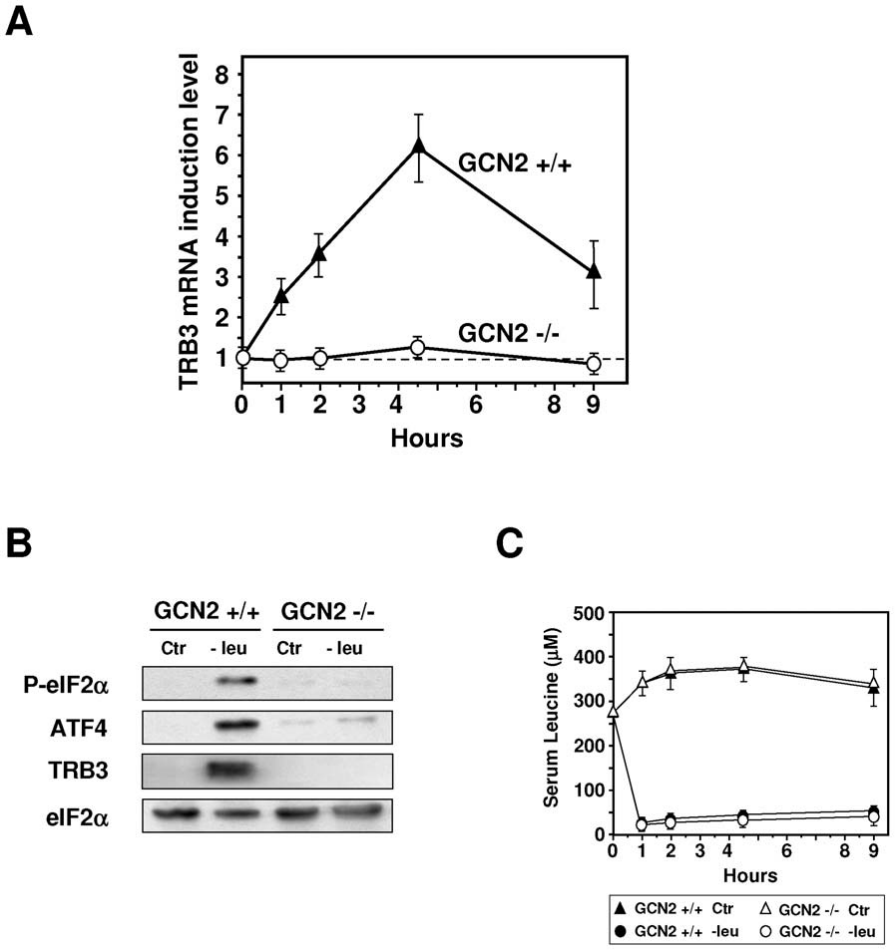
Role of GCN2 in the induction of TRB3 expression in the liver. GCN2 +/+ and GCN2 −/− mice were fed either control (Ctr) or leucine-deficient diets (−leu) prior to liver mRNA levels measurement. (A) Total hepatic RNA from GCN2 +/+ and GCN2 −/− mice fed for the indicated time was analyzed for TRB3 mRNA content. The TRB3 mRNA induction level is defined as the ratio of the relative mRNA level of leucine-starved mice to that of control mice. (B) Liver protein extracts from GCN2 +/+ or GCN2 −/− mice fed the indicated chow for 2 h were prepared and immunoblots against eIF2α phosphorylated on serine 51, ATF4, TRB3 and eIF2α were performed. (C) Plasma leucine levels (µM) of GCN2 +/+ or GCN2 −/− mice fed the indicated chow for 0–9 h. Each animal experiment was repeated three times with four animals in each group in order to confirm the reproducibility of the results.

To explore *in vivo* the role of GCN2 in the induction of TRB3 expression, we examined the kinetic of the increase in TRB3 mRNA in the liver of both knockout (GCN2 −/−) and wild-type (GCN2 +/+) mice fed a control or leucine-deficient diet (Figure 5A). In GCN2 +/+ mice, TRB3 mRNA was increased 2.5-fold, 1 h after the beginning of the meal and reached a maximum level (6-fold) after 4 h30. In contrast, lack of GCN2 resulted in a complete loss of TRB3 mRNA inducibility. Protein analysis confirms that the lack of GCN2 affected the phosphorylation of eIF2α as well as the level of TRB3 and ATF4 expression (Figure 5B). Serum leucine levels were decreased to similar levels by the leucine-deficient diet in both genotypes thereby indicating an altered response to amino acid deficiency in mice lacking GCN2 activity (Figure 5C). Thus, in the mice liver, GCN2 is essential for induction of the *TRB3* gene in response to a leucine-deficient diet.

### Identification of factors involved in *TRB3* AARE regulation in liver tissues

**Figure 6:**
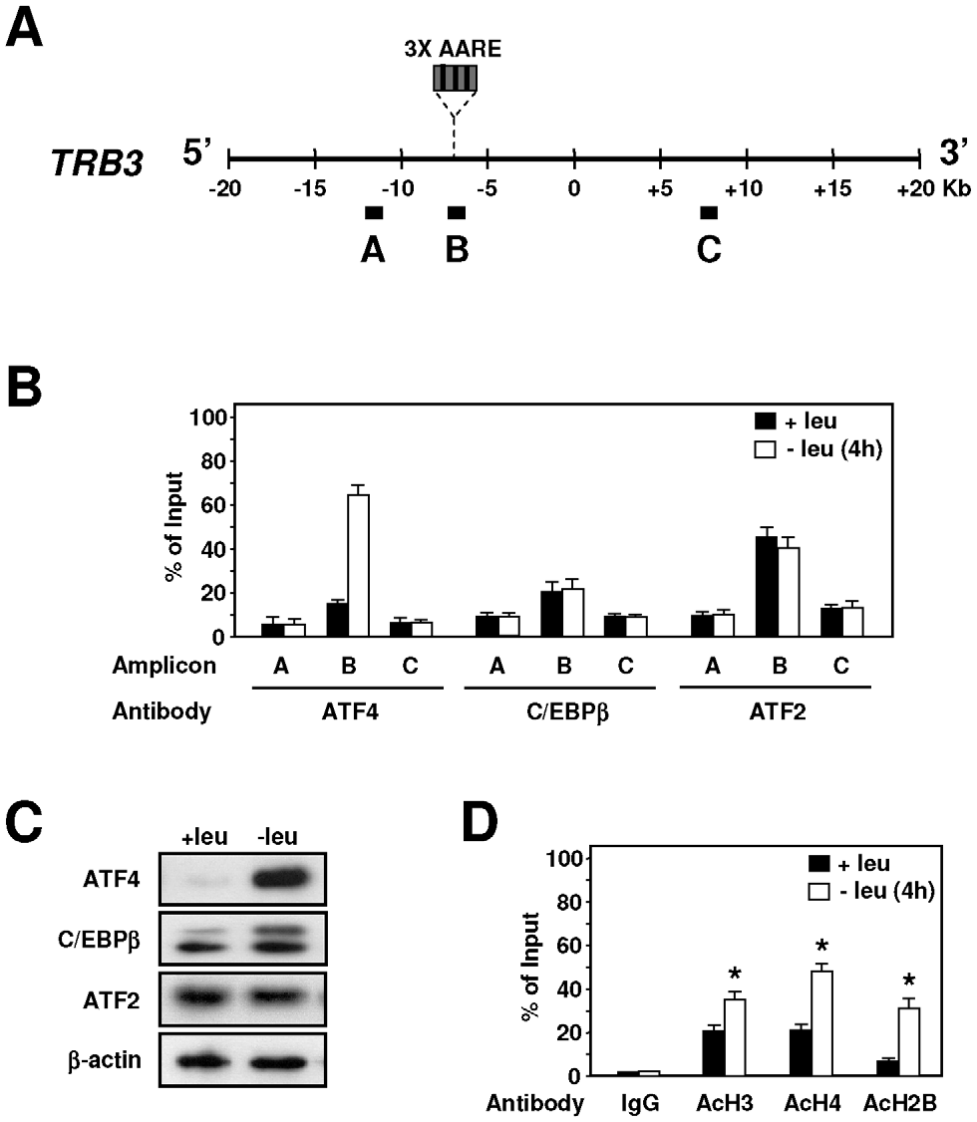
Transcription factor binding to *TRB3* AARE in response to leucine starvation. (A) Scheme of the human *TRB3* gene indicating the different amplicons produced for the ChIP analysis: A (−11952 to −11800 bp), B (−7210 to −7001 bp) and C (+8166 to +8384 bp). The AARE is boxed in grey. (B) HepG2 cells were incubated 4 hours either in control (+leu) or leucine-free medium (−leu) and harvested. ChIP analysis was performed as described under “Materials and Methods” using antibodies specific for ATF4, C/EBPβ and ATF2 and different sets of primers to produce amplicon A, B or C. Data were plotted as the percentage of antibody binding versus the amount of PCR product obtained using a standardized aliquot of input chromatin. (C) HepG2 cells were incubated either in control (+leu) or leucine-free medium (−leu) and harvested after 4 hours and western blot analysis of ATF4, C/EBPβ, ATF2 or β-actin was performed. (D) The experiment described in (B) was also performed using antibodies specific for acetylated H3, acetylated H4 and acetylated H2B. * Statistical significance (*P*<0.05) of the leucine-deprived values compared with the values in the control medium-incubated cells.

It has been previously demonstrated that in amino acid-starved cells, a multiproteic complex is bound to the AARE sequences including a number of regulatory proteins like ATF4 [7], [12], ATF2 [10] and C/EBPβ [14]. To determine whether these factors bind the AARE sequences whithin the *TRB3* promoter, HepG2 cells were incubated in control or leucine free medium for 4 h and ChIP assays were performed with primer sets covering either the 5′ region (amplicon A), the AARE (amplicon B) or the 3′ region (amplicon C) of the *TRB3* gene (Figure 6A). The results show an increase in ATF4 binding to the AARE following 4 h of leucine deprivation whereas the binding of ATF2 and C/EBPβ remained constitutive (Figure 6B). Furthermore, binding of ATF4, ATF2 and C/EBPβ was not detected in the 5′ and 3′ regions of *TRB3* confirming that these factors bind specifically to the AARE. Protein analysis confirms that ATF4 and C/EBPβ expression increased in leucine-starved HepG2 cells as described previously [37] while ATF2 level remained unchanged (Figure 6C). To test the influence of leucine deprivation on the modification of chromatin structure, acetylation of histones was analyzed. The abundance of acetylated histones H3, H4 and H2B significantly increased in leucine-deprived HepG2 cells compared to the control (Figure 6D). This increase was observed in the AARE as well as in the 5′ and 3′ regions of *TRB3* indicating that the change in histone acetylation is propagated in the vicinity of the AARE (data not shown).

**Figure 7:**
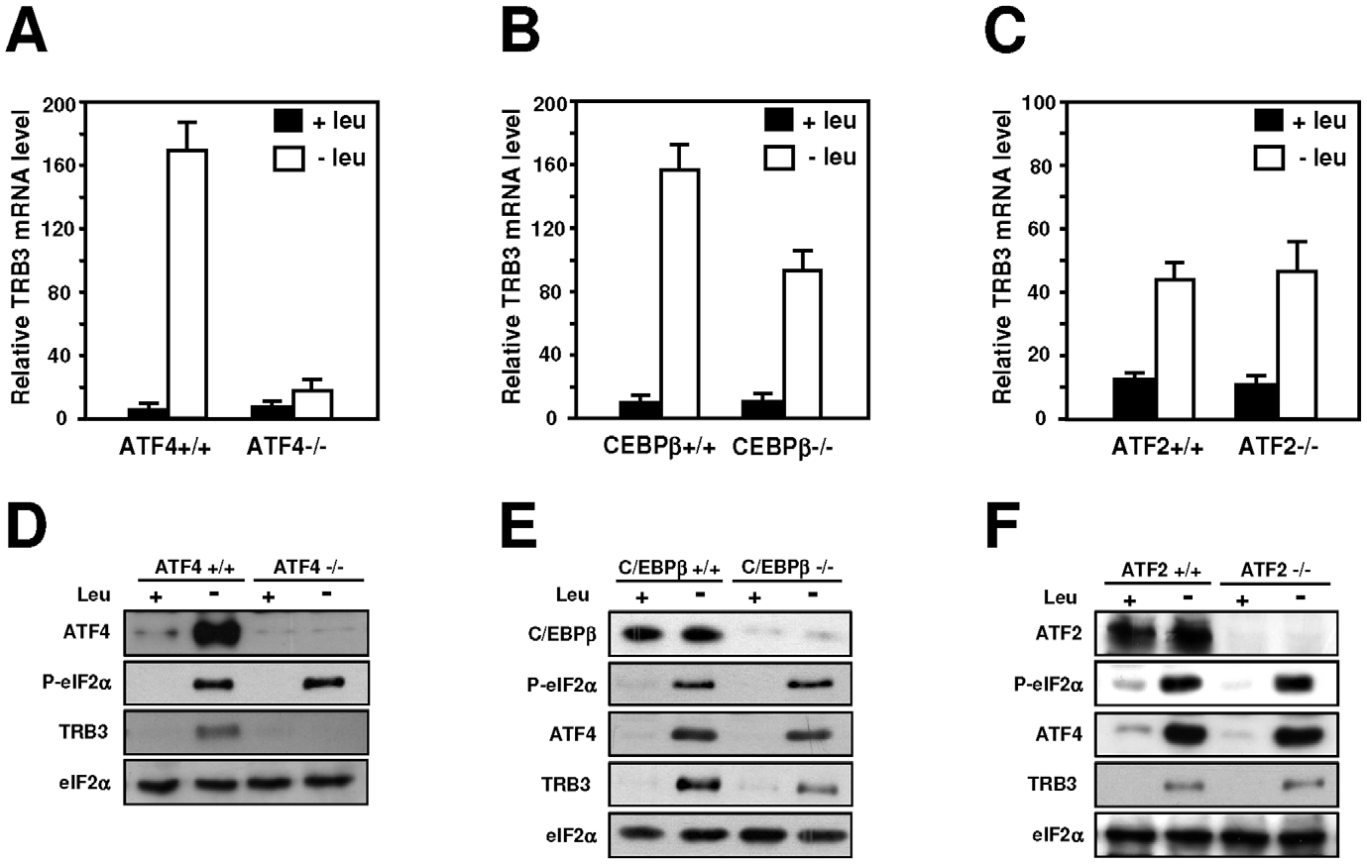
Role of ATF4, C/EBPβ and ATF2 in the induction of transcription in response to leucine starvation. Wild-type (+/+) cells and cells deficient (−/−) for ATF4 (A), C/EBPβ (B) or ATF2 expression (C) were incubated 4 hours either in control (+leu) or leucine-free medium (−leu) and total RNA was analyzed for *TRB3* mRNA content as described under “Materials and Methods”. Protein extracts from (D) ATF4 +/+ and ATF4 −/− MEFs, (E) C/EBPβ+/+ and C/EBPβ−/− MEFs, (F) ATF2 +/+ and ATF2 −/− MEFs incubated either in control (+leu) or leucine-free medium (−leu) and harvested after 4 hours were prepared and immunoblots against ATF4, C/EBPβ, ATF2, TRB3, eIF2α phosphorylated on serine 51 or total eIF2α were performed as described under “Materials and Methods”.

To determine whether ATF4, ATF2 or C/EBPβ are required to mediate the TRB3 mRNA induction following leucine starvation, MEFs deficient in ATF4, ATF2 or C/EBPβ and their corresponding wild-type cells were used. TRB3 mRNA level was enhanced in wild-type MEF cells deprived of leucine. Lack of ATF4 abolished (Figure 7A) whereas lack of C/EBPβ only reduced the *TRB3* mRNA inducibility by leucine depletion (Figure 7B). In contrast, the increased expression level of TRB3 mRNA resulting from amino acid deprivation was not affected in ATF2−/− cells (Figure 7C). Protein analysis shows that lack of ATF4 resulted in a complete loss of the induction of TRB3 expression (Figure 7D) confirming that ATF4 is required for the transcription activation of *TRB3* following leucine starvation. By contrast, leucine deprivation induced phosphorylation of eIF2α, ATF4 and TRB3 expression in C/EBPβ−/− (Figure 7E) and ATF2−/− MEFs (Figure 7F) providing evidence that ATF2 and C/EBPβ are not involved in amino acid regulation of *TRB3*.

**Figure 8:**
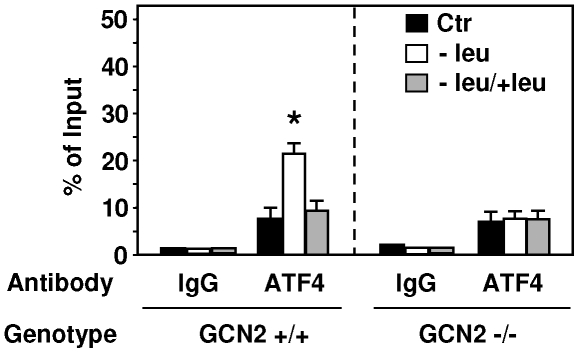
ATF4 binding to *TRB3* AARE in the liver of leucine-deprived mice. GCN2 +/+ and GCN2 −/− mice were fed for 2 h either the control (Ctr) or leucine-deficient diets (−leu) or refed for 2 h a control diet after a 2 h-consumption of the leucine-deficient diet (−leu/+leu) and then ATF4 binding associated with the *TRB3* AARE was monitored in liver extracts by ChIP assays. PCR products amplifying the AARE-containing promoter of the *TRB3* gene were assayed by qPCR as described under “Materials and Methods”. Data were plotted as the percentage of antibody binding versus the amount of PCR product obtained using a standardized aliquot of input chromatin. Each point represents the mean value of three independent experiments, and the error bars represent the standard error of the means. * Statistical significance (*P*<0.05) of the leucine-deprived values compared with the values in the control medium-incubated cells.

The results described above highlight the key role of ATF4 in the transcriptional activation of *TRB3* following amino acid starvation in HepG2 cells. To examine whether the binding of ATF4 to *TRB3* AARE is also increased in the liver of mice fed either the leucine-deficient or the control diet for 2 h, ChIP experiments were performed (Figure 8). The results show an increase in ATF4 binding to the AARE in the liver of wild type mice (GCN2 +/+) in response to leucine starvation and are consistent with those described in HepG2 cells. A marked decrease in ATF4 binding was observed when mice are refed 2 h with a control diet after leucine deprivation. As expected the increase in ATF4 binding to the *TRB3* AARE was lost in the liver of knockout (GCN2 −/−) mice fed a leucine-deficient diet. Taken together, these results demonstrate that ATF4 binding to the AARE, is a key event in the transcriptional induction of *TRB3* in the liver of mice fed with a leucine-deficient diet.

## Discussion

In order to adapt to an amino acid imbalanced diet, mammalian cells regulate expression of specific genes involved in transport, intermediary metabolism, oxidative stress and energy metabolism. In cultured cell lines, hundreds of amino acid-responsive genes have been identified by microarray analysis [36], [38], [39], although few of them have been extensively investigated with regard to transcriptional mechanisms. In the last decade, significant progress has been achieved in the understanding of molecular mechanisms involved in the control of gene transcription following amino acid limitation in cultured cells [1], [5]. However, so far no results concerning transcriptional mechanisms was obtained directly in mammalian tissues.

In a mammalian organism, the control of gene transcription differs in many aspects from that operating in cultured cells, and involves complex interactions between hormonal, neuronal and nutritional factors. The regulation of *TRB3* gene expression represents a new mechanistic model to investigate how the consumption of a leucine-deficient diet can activate gene transcription in mice liver. The experiments included in this report have led to the following novel observations. 1) *TRB3* is up-regulated in the liver of mice fed a leucine-deficient diet and this induction is quickly reversible. 2) Using transient transfection and chromatin immunoprecipitation approaches in hepatoma cells, we report the characterization of an AARE in the *TRB3* promoter and the binding of ATF4, ATF2 and C/EBPβ to this AARE sequence. 3) We provide also evidence that only ATF4 bound to the AARE plays a crucial role in the amino acid-regulated transcription of *TRB3*. 4) Using GCN2−/− mice and a chromatin immunoprecipitation approach, we demonstrate that the GCN2/eIF2α~/ATF4 pathway is essential for induction of the *TRB3* gene transcription in response to a leucine-deficient diet.

The 5′-upstream region of the human *TRB3* gene contains three identical tandemly arranged repeats each consisting of 33-bp. This promoter region was reported to contain an endoplasmic reticulum (ER) stress response element [17], [29]. The present results provide evidence that these 33-bp repeats are also responsible for the amino acid control of *TRB3* transcription. These DNA sequence can be called an AARE for the following reasons: (i) the 33-bp repeats can regulate a basal promoter in response to leucine (see Figure 3C) or some other essential amino acids starvation (data not shown); (ii) the sequence of the middle part of the 33-bp repeat (5′-TTTGCATCA-3′) differs from the AARE core sequence of the *CHOP* (5′-ATTGCATCA-3′), *SNAT2* (5′-ATTGCATCA-3′) and *ATF3* (5′-ATTGCATCA-3′) genes by only one nucleotide and from the AARE core sequence (5′-GTTTCATCA-3′) of the *ASNS* gene by 2 nucleotides (see Figure 3B); (iii) mutations affecting the core sequence of the 33-bp repeat result in a sharp decrease of amino acid responsiveness (Figure S1). In addition, the *TRB3* AARE confers a higher amino acid responsiveness than *CHOP* or *ATF3* AAREs.

As for the other AAREs described previously, the sequence of the *TRB3* AARE show some homology with the specific binding sites of the C/EBP and ATF/cAMP response element-binding protein transcription factors. Du *et al* [33] reported that C/EBPβ is involved in the induction of *TRB3* by insulin. In the context of gene regulation by amino acid starvation, C/EBPβ like ATF2, do not seem to be involved in the amino acid-regulated transcription although both factors bind to the *TRB3* AARE sequence. Using EMSA (electrophoresis mobility shift analysis), Örd *et al.* [29] demonstrated that ATF4 binds *in vitro* the *TRB3* AARE in response to ER stress. The present observations provide *in vivo* evidence for ATF4 binding to *TRB3* AARE in both amino acid-starved hepatoma cells and in the liver of mice fed a leucine-deficient diet. The effects of ATF4 on *TRB3* transcription in mice liver appears to be quickly reversible since marked decreases in both ATF4 expression and binding to AARE were observed when mice are refed 2 h a control diet after leucine deprivation. Therefore, *TRB3* can be quickly switched on or switched off in the liver of mammals according to the amino acid content of the diet. It is possible that other ATF4 interacting factors or co-factors should be involved in this regulatory process of transcription in the liver. These proteins remain to be identified.

At a physiological level, TRB3 is involved in the regulation of many biological functions in several tissues. By inhibiting Akt activation by insulin in liver [19] and acetyl–coenzyme A carboxylase (ACC) in adipose tissue [21], TRB3 has been found to modulate lipid and glucide metabolisms. Currently, the mechanisms involved in the regulation of TRB3 expression in liver and adipose tissue are not well understood. An increasing number of data provides arguments suggesting that TRB3 could be also involved in the control of amino acid and protein metabolism. It was shown that (i) TRB3 is a potent regulator of P70S6 kinase (S6K1) activation by insulin [40]; (ii) TRB3 is involved in the induction of autophagy by inhibiting the Akt/mTORC1 axis [41]; (iii) TRB3 inhibits ATF4-dependent transcription in response to amino acid starvation [16], [24]; (iv) We show in this work that GCN2 activation and ATF4 binding to the *TRB3* AARE, are key events in the up-regulation of *TRB3* transcription in the liver of mice fed a leucine-deficient diet. Thus, it is likely that TRB3 could participate in the control of amino acid homeostasis. However its precise role in the control of amino acid metabolism remains to be investigated.

It is now clear that when animals are presented with a diet devoid of a single essential amino acid, the GCN2/eIF2α pathway is activated in several tissues. Following the consumption of such diet, the decline in the amino acid blood levels is correlated with the GCN2-dependent phosphorylation of eIF2α and a sharp food intake inhibition [42], [43]. Previous studies have involved the anterior piriform cortex in sensing blood amino acid levels and in initiating the food intake response [44]. GCN2 kinase-deficient mice illustrated that recognition of an amino acid imbalanced-diet by the brain requires uncharged tRNA sensing by GCN2 [42], [43]. However, the role of ATF4 in this process remains to be demonstrated. It seems likely that in most tissues, following activation of the GCN2/eIF2α/ATF4 pathway, a highly coordinated time-dependent program of molecular events should take place, leading to the fine transcriptional regulation of specific target genes. Our results show for the first time that, in the liver, amino acid control *TRB3* transcription through GCN2 activation and subsequent ATF4 binding to AARE sequences. The *TRB3* transcription is also up-regulated in other tissues following the consumption of a leucine-deficient diet, such as the intestine (data not shown). Nevertheless, other ATF4-target genes remain to be identified in these tissues.

The idea that amino acids can regulate gene transcription in mammalian tissues through the GCN2/eIF2α/ATF4 pathway is now established. The molecular basis of gene regulation by dietary protein intake is an important field of research for studying regulation of physiological functions of individuals living under condition of restricted, imbalanced, or excessive food intake. Beyond gaining a basic understanding of the amino acid control of biological mechanisms, the characterization of how these processes contribute to the occurence of various diseases represents an important field of investigation in molecular nutrition.

## Materials and Methods

### Ethics Statement

Maintenance of the mice and all experiments were conducted according to the guidelines formulated by the European Community for the use of experimental animals (L358-86/609/EEc) and were approved by the Institut National de la Recherche Agronomique (INRA-France). INRA animal facilities were approved by the french veterinary department (C634514).

### Animals

The generation of GCN2-null mice has been described in detail elsewhere [42]. Mice were maintained in our animal facility in a temperature-controlled room (22±1°C) on a 12:12 h light-dark cycle. They were provided free access to commercial rodent chow (pellets A03 from Safe, Augy, France) and tap water prior to the experiment. Experimental diets were manufactured in our institute facilities (INRA, Unité de Préparation des Aliments Expérimentaux, Jouy-en-Josas, France). They contained 20% free L-amino acids as the sole protein source calculated on the base of the lactoserum amino acid composition. The control (Ctr) diet contained 20 amino acids including leucine. The leucine-devoid (-leu) diet had exactly the same composition except for branched-chain amino acids. The lack of nitrogen resulting from leucine deficiency was adjusted with alanine. Furthermore, isoleucine and valine levels were reduced in order to keep the blood concentration of these amino acids constant after eating the leucine-devoid diet. At the beginning of the feeding experiment, eight to ten-week-old male mice were first acclimated to control diet for 7 days and to overnight starvation. On the morning of day of experiment, mice were randomly assigned to either control diet group or (-)leu diet group. They had free access to these diets for 1 h, 2 h, 4 h30 or 9 h until they were killed by pentobarbital overdose. Liver were isolated, snap frozen and stored at -80°C for future analysis. Each animal experiment was repeated three times with four animals in each group in order to confirm the reproducibility of the results.

### Plasma amino acid analysis

Blood samples were drawn in the aorta of anesthetized mice. Plasma samples were treated with sulfosalicylic acid and thiodiglycol. Free amino acids proportions were determined using an ion-exchange liquid chromatography followed by post-column detection with ninhydrine (Bio-Tek system). The internal standard, norleucine, allowed the evaluation of sample treatment efficiency in order to correct the crude values.

### Cell culture and treatment conditions

HepG2 cells (from ATCC) and mouse embryonic fibroblasts (MEF) were cultured at 37°C in Dulbecco's modified Eagle's medium F12 (DMEM F12) (Sigma) containing 10% fetal bovine serum. When indicated, DMEM F12 lacking leucine, lysine, methionine or glutamine (DMEM F12 Base) (Sigma) was used. In all experiments involving amino acid starvation, 10% dialyzed calf serum was used. Wild-type MEFs and MEFs deficient in GCN2 [45], PERK [46], ATF4 [4] and in C/EBPβ [47] were kindly given by Dr. D. Ron (Skirball Institute of Biomolecular Medicine, New York). MEFs deficient in ATF2 were a gift of Dr. N. Jones (Paterson Institute for Cancer Research) [48].

### Analysis of gene expression using real time RT-PCR

Total RNA was prepared using a RNeasy mini kit (Qiagen) and treated with DNase I, Amp Grade (InVitrogen) prior to cDNA synthesis. RNA integrity was electrophoretically verified by ethidium bromide staining. RNA (0.5 µg) was reverse transcribed with 100 U of Superscript II plus RNase H^-^ Reverse Transcriptase (InVitrogen) using 100 µM random hexamer primers (Amersham Biosciences), according to the manufacturer's instructions. To measure the relative amount of human and mouse TRB3 mRNA, primers used were the following: h-TRB3 (forward primer, 5′-TGGTACCCAGCTCCTCTACG-3′; reverse primer, 5′-GACAAAGCGACACAGCTTGA-3′) and m-TRB3 (forward primer, 5′-CAGGAAGAAACCGTTGGAGTT-3′; reverse primer, 5′-TTGCTCTCGTTCCAAAAGGA-3′). All the primers yielded PCR products of 200 bp. To control for RNA quality and cDNA synthesis, human and mouse β-actin mRNA were also amplified with the following primers: h-β-actin (forward primer, 5′-TCCCTGGAGAAGAGCTACGA-3′; reverse primer, 5′- AGCACTGTGTTGGCGTACAG-3′) and m-β-actin (forward primer, 5′-AAGGAAGGCTGGAAAAGAGC-3′; reverse primer, 5′-TACAGCTTCACCACCACAGC-3′). To measure the transcriptional activity from the *TRB3* gene, oligonucleotides derived from *TRB3* intron 2 and exon 3 were used to measure the short-lived unspliced transcript (hnRNA, heterogeneous nuclear RNA). This procedure for measuring transcriptional activity is based on that described by Lipson and Baserga [34]. The TRB3 primers for amplification were: forward primer, 5′-GAGTCCCCAGCTGTGCTAAC-3′; reverse primer, 5′-GTCCGAGTGAAAAAGGCGTA-3′. Quantification involved the use of standard curves that had been prepared with plasmids containing specific sequences of each gene. We cloned all the PCR products into the pGEM-T easy vector (Promega) according to the manufacturer's instructions. For the construction of standard curves, pGEM-T easy plasmids were prepared as 10-fold serial dilution in water, from 4 ng to 0.4 pg. PCR was carried out using a LightCycler™ System (Roche) as described previously [12]. LightCycler quantification software (version 3.5) was used to compare amplification in experimental samples during the log-linear phase to the standard curve from the dilution series of control plasmids. Relative results were displayed in nanograms of target gene per 100 nanograms of β*-actin*. Each experiment was repeated three times to confirm the reproducibility of the results.

### Plasmid constructions

All constructs containing deletions or mutations in the *TRB3* promoter have been previously described [29]. The numbering system in the *TRB3* promoter has been established according to the identification of the transcription start sites [49]. 2XAARE-*CHOP*-TK-LUC and 2XAARE-*ATF3*-TK-LUC plasmids was generated as previously described [10], [50]. 2XAARE-*TRB3*-TK-LUC construct was generated by inserting M*lu*I-X*ho*I doubled stranded oligonucleotides containing two iterations of the *TRB3* AARE sequence (−7131 to −7033) into the M*lu*I-X*ho*I sites of TATA-TK-LUC [10].

### Transient transfection and luciferase assay

Cells were plated in 12 well-dishes and transfected by the calcium phosphate co precipitation method as described previously [10]. One µg of luciferase plasmid was transfected into the cells along with 0.05 µg of pCMV-ßGal, a plasmid carrying the bacterial ß-galactosidase gene fused to the human cytomegalovirus immediate-early enhancer/promoter region, as an internal control. Cells were then exposed to the precipitate for 16 h, washed twice in phosphate buffered saline (PBS), and then incubated with DMEM F12 containing 10% fetal bovine serum. Two days after transfection, cells were harvested in 100 µl of lysis buffer (Promega) and centrifuged at 13,000×g for 2 min. Twenty µl of the supernatant were assayed for luciferase activity (YELEN, Ensue La Redonne, France). For all the transfection experiments presented, a plasmid pCMV-βGAL was used as an internal control. ß-Galactosidase activity was measured as described previously [10]. Relative luciferase activity was given as the ratio of relative luciferase unit/relative ß-Gal unit. All values are the means calculated from the results of at least three independent experiments performed in triplicate.

### Antibodies

The following antibodies were purchased from Santa Cruz Biotechnology, Inc (Santa Cruz, CA): ATF2, sc-187; ATF4, sc-200; C/EBPβ, sc-150; β-actin, sc-7210. The TRB3 antibody (ST 1032) was obtained from Calbiochem and the P-eIF2α antibody (catalog no. 1090-1) was from Epitomics (Burlingame, CA). Acetylated histone H3, 06-599 (recognizes acetylated H3 at Lys-9 and Lys-14) and acetylated histone H4, 06-866 (recognizes acetylated H4 at Lys-5, -8, -12 and -16) antibodies were purchased from Upstate Biotechnology (Charlottes-ville, VA). Acetylated histone H2B (recognizes acetylated H2B at Lys-12 and Lys-15) antibody was from Abcam (Cambridge, UK).

### Nuclear extracts

Nuclear extracts from mouse liver cells were prepared as described by Sierra [51].

### Immunoblot analysis

To detect eIF2α and its phosphorylated form, liver cells were lysed in radioimmune precipitation assay buffer (50 mM Tris-HCl, pH 7.4, 150 mM NaCl, 1% Triton X-100, 0.1% SDS, 50 mM NaF, 2 mM Na_3_VO_4_, 100 nM acid okadaic, 25 mM β-glycerophosphate, 1 mM phenylmethylsulfonyl fluoride, protease inhibitor cocktail from Sigma). To determine ATF4 and TRB3 proteins in liver samples, nuclear extracts were prepared. Total or nuclear proteins were resolved by SDS-polyacrylamide gel electrophoresis and transferred onto a Hybond-P PVDF membrane (Amersham Biosciences). Membranes were blocked for 1 h at room temperature with a solution of 5% nonfat milk powder in TN (50 mM Tris-HCL, pH 8.0, 150 mM NaCl, 0.1% Tween-20). The blots were then incubated with primary antibody in blocking solution overnight at 4°C. Antibodies were diluted according to the manufacturer's instructions. The blots were washed three times in TN and incubated with horseradish peroxidase-conjugated goat anti-rabbit IgG (1:5000) (Santa Cruz, CA) in blocking buffer for 1 h at room temperature. After three washes, the blots were developed using the enhanced chemiluminescence (ECL) detection system (Amersham Biosciences).

### Chromatin immunoprecipitation analysis (ChIP)

ChIP analysis was performed according to the protocol of Upstate Biotechnology, Inc. (Charlottesville, VA) with minor modifications. HePG2 cells were seeded at 1×10^6^/100-mm dish with DMEM F12 and grown for 24 h. Cells were transferred to fresh DMEM F12 12 h before transfer to either complete DMEM F12 or DMEM F12 lacking leucine for the time period indicated in each figure. For liver, fresh samples (900 mg) were chopped in small pieces and rinsed with fresh ice-cold phosphate buffered saline, pH 7.5 (PBS). Protein-DNA was cross-linked by adding formaldehyde directly to the culture medium or to liver samples to a final concentration of 1% and then stopped 15 min later by the addition of glycine to a final concentration of 0.125 M. For mice liver samples, nuclear extracts were then prepared. Cross-linked chromatin was sonicated using a Vibra cell sonicator (Biobloc Scientific Technology) for ten bursts of 1 min at power 2 with 1-min cooling on ice between each burst to obtain DNA fragments of an average of 400 bp. Liver or cells extracts were incubated with 5 µg of antibody. A rabbit anti-chicken IgG was used as the nonspecific antibody control. The antibody-bound complex was precipitated by protein A-Agarose beads (Upstate Biotechnology). The DNA fragments in the immunoprecipitated complex were released by reversing the cross-linking overnight at 65°C and purified using a phenol/chloroform extraction and ethanol precipitation. Real-time quantitative PCR was performed by using a LightCycler (Roche) and a SYBR-Green-I-containing PCR mix (Qiagen), following the recommendations of the manufacturer. The immunoprecipitated material was quantified relative to a standard curve of genomic DNA. Primers used for the human *TRB3* promoter: *hTRB3* amplicon A, 5′-AAGAGAAAAGCAGCCTTCTGG-3′ and 5′-AGCGAGGAAAAGAATGGTGA-3′; *hTRB3* amplicon B (AARE), 5′- GCGGATGCAGAGGAGAGA-3′ and 5′- CACTTCCGCTGCGAGTCT-3′; *hTRB3* amplicon C, 5′-CCCATGTCCCAGGAAGAAG-3′ and 5′-AGTCCTGGAAGGGGTAGTGG-3′. The set of primers used for the analysis of the mouse *TRB3* promoter were 5′-GGGCGGGTCACAGATGGTGC-3′ and 5′-GACCGCCGCCAGCCTAACTG-3′. The reactions were incubated at 95°C for 15 min to activate the polymerase, followed by amplification at 95°C for 15 s, 59°C for 20 s (human primers), 68°C for 20 s (mouse primers) and 72°C for 20 sec for 45 cycles. After PCR, melting curves were acquired by stepwise increases in the temperature from 65 to 95°C to ensure that a single product was amplified in the reaction. The results are expressed as the percentage of antibody binding versus the amount of PCR product obtained using a standardized aliquot of input chromatin. Samples are the means from at least three independent immunoprecipitations.

## Supporting Information

Figure S1 Responsiveness of the wild-type and mutant 33-bp repeats to leucine starvation. HepG2 cells were transfected with luciferase reporter constructs [29] driven by the wild-type (wt) 33-bp repeat or mutant 33-bp repeats (mut-5′, mut1-AARE core, mut2-AARE core, mut-3′). The AARE core sequence within the nucleotide sequence of the 33-bp repeat is boxed and mutated nucleotides are in italic lower-case letters. Twenty-four hours after transfection, cells were incubated for 24 h in DMEM F12 (+leu) or in DMEM F12 lacking leucine (−leu) and then were harvested for preparation of cell extracts and determination of luciferase (LUC) activity. Relative LUC activities were determined as described in “Materials and Methods”. The relative fold induction, defined as the ratio of the relative LUC activity of leucine-starved cells to unstarved cells, is indicated in parentheses to the right of the bars. Each data represents the mean of at least three independent experiments performed in triplicate.
(TIF)
