# Supplementary figures and images for: Amino Acid Availability Controls TRB3 Transcription in Liver through the GCN2/eIF2α/ATF4 Pathway
Source: PLoS One. 2010 Dec 21;5(12):e15716. doi: 10.1371/journal.pone.0015716 (PMC3006201; doi:10.1371/journal.pone.0015716)

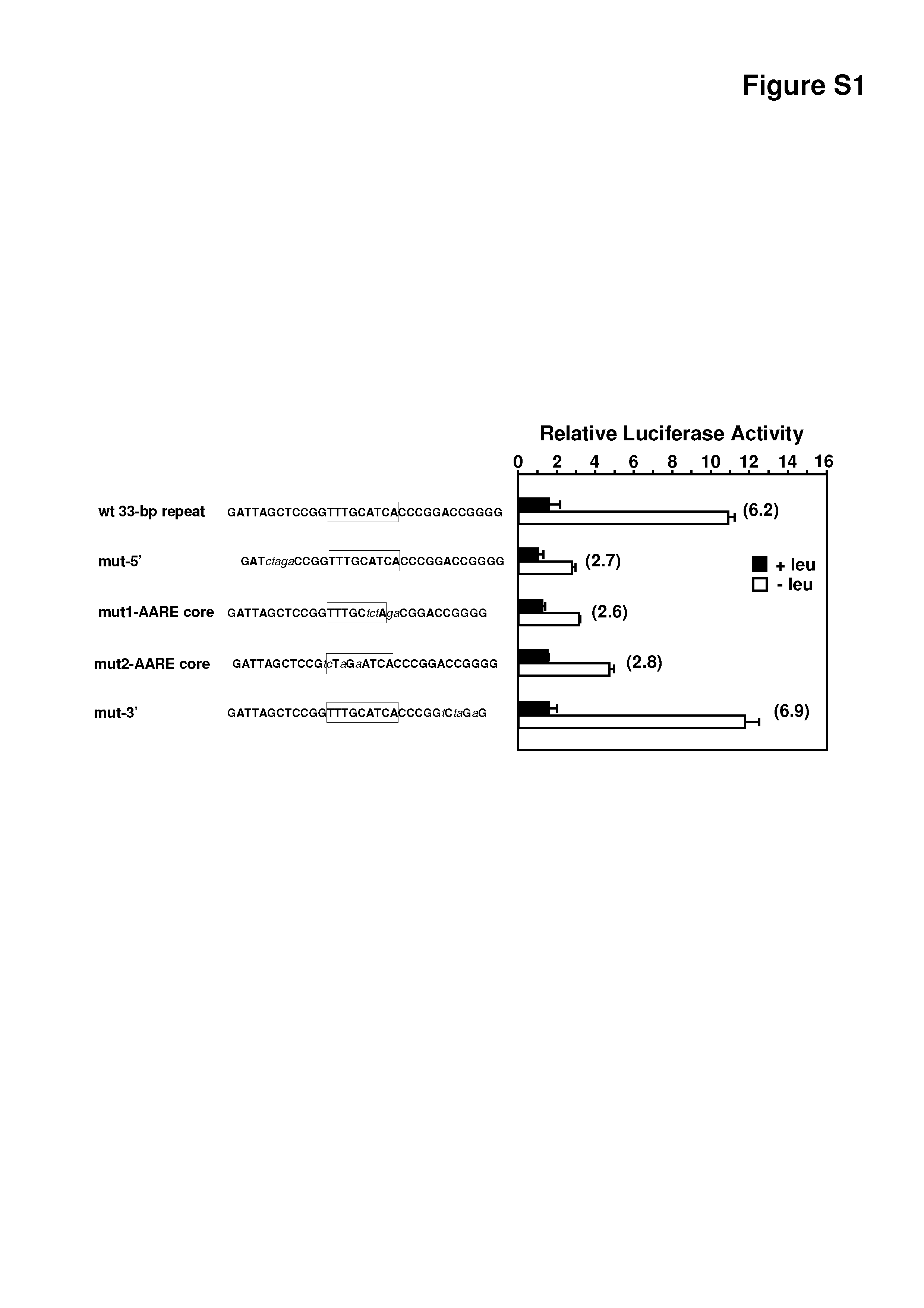

Supplement: Figure S1 — Responsiveness of the wild-type and mutant 33-bp repeats to leucine starvation. HepG2 cells were transfected with luciferase reporter constructs [29] driven by the wild-type (wt) 33-bp repeat or mutant 33-bp repeats (mut-5′, mut1-AARE core, mut2-AARE core, mut-3′). The AARE core sequence within the nucleotide sequence of the 33-bp repeat is boxed and mutated nucleotides are in italic lower-case letters. Twenty-four hours after transfection, cells were incubated for 24 h in DMEM F12 (+leu) or in DMEM F12 lacking leucine (−leu) and then were harvested for preparation of cell extracts and determination of luciferase (LUC) activity. Relative LUC activities were determined as described in “Materials and Methods”. The relative fold induction, defined as the ratio of the relative LUC activity of leucine-starved cells to unstarved cells, is indicated in parentheses to the right of the bars. Each data represents the mean of at least three independent experiments performed in triplicate. (TIF) [file pone.0015716.s001.tif]
